# Is Early Injection-Site Reaction Associated With the Efficacy of Romosozumab?

**DOI:** 10.7759/cureus.109243

**Published:** 2026-05-19

**Authors:** Takaomi Kobayashi, Tomonori Kobayakawa, Tadatsugu Morimoto, Yukio Nakamura

**Affiliations:** 1 Orthopaedic Surgery, Faculty of Medicine, Saga University, Saga, JPN; 2 Liberal Arts, Faculty of Healthcare and Welfare, Saitama Prefectural University, Saitama, JPN; 3 Preventive Medicine, Faculty of Medicine, Saga University, Saga, JPN; 4 Education and Research Center for Community Medicine, Faculty of Medicine, Saga University, Saga, JPN; 5 Orthopaedic Surgery, Kobayakawa Orthopedic and Rheumatologic Clinic, Shizuoka, JPN; 6 Osteoporosis, Locomotive Syndrome, Joint Disease Center, Aichi Medical University, Nagakute, JPN; 7 Orthopaedic Surgery, Aichi Medical University, Nagakute, JPN

**Keywords:** bone mineral density, immunoporosis, injection-site reaction, romosozumab, women

## Abstract

Introduction

Injection-site reactions (ISRs) associated with romosozumab may indicate immune activation that influences bone mineral density (BMD) response, aligning with the concept of “immunoporosis.” This study aimed to investigate whether early ISRs following romosozumab administration are associated with greater increases in BMD in women with primary osteoporosis.

Methods

We conducted a sub-analysis of a previously reported prospective cohort study involving 181 untreated Japanese women aged 52-100 years with primary osteoporosis who began a 12-month course of romosozumab. Patients were categorized into an ISR group (n=44) and a non-ISR group (n=137) based on the occurrence of ISRs within the first six months of treatment. Percent changes in lumbar spine (LS), total hip (TH), and femoral neck (FN) BMD at six and 12 months were compared between groups using Student's t-test or Fisher's exact test.

Results

The ISR group exhibited significantly greater increases in LS BMD compared to the non-ISR group at six months (12.8% vs. 9.5%, p=0.039) and 12 months (18.0% vs. 14.4%, p=0.046). In contrast, no significant differences were detected between groups in TH BMD at six months (3.5% vs. 2.9%, p=0.647) or 12 months (6.7% vs. 5.5%, p=0.330), or in FN BMD at six months (4.0% vs. 3.5%, p=0.588) or 12 months (6.7% vs. 5.4%, p=0.326).

Conclusion

ISR could serve as a no-cost and clinically observable indicator associated with LS BMD response to romosozumab that requires no additional testing. Further studies are needed to validate this hypothesis and clarify the underlying mechanisms.

## Introduction

Romosozumab is a humanized monoclonal antibody that exerts dual effects on bone metabolism by promoting bone formation and inhibiting bone resorption through sclerostin inhibition and subsequent activation of Wnt signaling [1,2]. Clinical studies have demonstrated that 12-month romosozumab treatment significantly increases bone mineral density (BMD) at the lumbar spine (LS), total hip (TH), and femoral neck (FN), and reduces fracture risk [2-6], particularly in women with primary osteoporosis [7-9]. Recently, various potential predictors of romosozumab efficacy have been reported, including procollagen type 1 N-terminal propeptide (P1NP) [10-16], tartrate-resistant acid phosphatase isoform 5b (TRACP-5b) [14-16], estimated glomerular filtration rate (eGFR) [14,15], prior use and duration of antiresorptive therapy [15-17], and LS BMD [14,15]. However, clinically observable indicators of treatment response remain unexplored.

Injection-site reactions (ISRs)-such as erythema, swelling, or pain-are among the most common and well-recognized adverse events associated with romosozumab [2,18], reflecting localized immune activation [2-6]. These ISRs may potentially reflect systemic immune activation, as romosozumab’s mechanism of sclerostin inhibition and Wnt signaling activation modulates immune function by influencing immune cell differentiation and inflammatory responses [19-26]. Interestingly, systemic immune status is linked to bone metabolism, as highlighted by the concept of “immunoporosis,” which arises from the field of osteoimmunology [27,28]. Although direct evidence linking ISRs and skeletal response to romosozumab remains limited, concepts from osteoimmunology and “immunoporosis” suggest a possible interaction between immune activation and bone metabolism. Based on this, we hypothesized that early ISRs-occurring within the first six months of treatment-could serve as a clinically observable indicator of a favorable response to romosozumab.

Therefore, the primary objective of this study was to investigate whether the occurrence of ISRs within the first six months of romosozumab treatment is associated with greater subsequent gains in LS, TH, and FN BMD among women with primary osteoporosis. Secondary objectives included evaluating the association between ISRs and changes in bone turnover markers, including P1NP and TRACP-5b, during treatment. Because romosozumab-induced BMD changes are typically observed within the first year of treatment, we focused on ISRs occurring during the initial six months to evaluate their potential association with subsequent treatment response. This study is a secondary analysis of a previously reported prospective cohort study [18], which evaluated the real-world efficacy and safety of romosozumab in Japanese patients with osteoporosis. While the previous study focused on overall treatment outcomes and adverse events, the present study specifically investigates whether early injection-site reactions are associated with subsequent increases in bone mineral density using the same cohort.

## Materials and methods

Study design

We conducted a sub-analysis of a previously reported prospective cohort study [18] at our clinic and four collaborating institutions. The study included Japanese female patients with primary osteoporosis at high risk of fracture, as defined by the World Health Organization [29], who initiated 12-month romosozumab treatment between March 2019 and December 2021. Romosozumab was administered monthly at the standard approved dose of 210 mg for 12 months. A total of 184 patients were initially considered potentially eligible. Of these, three were excluded due to lack of evaluable data, leaving 181 patients for analysis.

All patients were recommended to receive calcium carbonate and vitamin D supplementation as appropriate. Those already receiving active vitamin D therapy were instructed to continue it throughout the study period.

This study analyzed data that had been previously collected and used in a prior study [18]. The protocol of this study was approved by the ethics committee of Chutoen General Medical Center (approval numbers: 3004231005). All participants provided written informed consent before enrollment. The study was conducted in accordance with the ethical principles of the Declaration of Helsinki.

Injection-site reaction

ISR was defined as the occurrence of localized symptoms, such as erythema, swelling, pain, or warmth at or around the injection site, lasting for two days or more, as reported by patients or observed during clinical visits [18]. Standardized questionnaires or severity grading systems were not used. Patients who experienced any ISR within the first six months of romosozumab treatment were categorized into the ISR group; those without ISR during this period were placed in the non-ISR group. The six months were selected to evaluate the association between ISR and subsequent changes in BMD at 12 months.

Bone mineral density

LS, TH, and FN BMD were measured at baseline and at six and 12 months using identical Prodigy Fuga DXA systems (GE Healthcare, Madison, WI, USA), with measurements performed by experienced technicians [7]. We calculated percent changes in LS, TH, and FN BMD from baseline to six and 12 months by using the formula: Percent change = [(BMD at follow-up - BMD at baseline) / BMD at baseline] × 100.

Bone turnover markers

At baseline, six months, and 12 months, bone turnover markers [i.e., P1NP and tartrate-resistant acid phosphatase isoform 5b (TRACP-5b)] were measured. Measurement of P1NP and TRACP-5b levels was performed using enzyme immunoassay and chemiluminescent enzyme immunoassay [7]. We calculated percent changes in these markers as follows: Percent change = [(marker level at follow-up - marker level at baseline) / marker level at baseline] × 100.

Statistical analysis

Several variables were compared between the ISR and non-ISR groups. Continuous variables were presented as mean±standard deviation and compared by using Student’s t-test. Categorical variables were expressed as numbers (percentages) and compared by using Fisher’s exact test. We investigated baseline characteristics, including age (years), body mass index (kg/m2), BMD (g/cm2), eGFR (mL/min/1.73 m2), serum albumin (g/dL), serum-corrected calcium (mg/dL), 25-hydroxyvitamin D (25OHD, ng/mL), P1NP (μg/L), TRACP-5b (mU/dL), prior vertebral fracture (yes/no), and non-vertebral fracture (yes/no). Outcomes of interest included percent changes in LS, TH, and FN BMD, as well as P1NP and TRACP-5b levels from baseline to six and 12 months.

Sensitivity analyses were performed using linear mixed models to test the robustness of the association between ISR within the first six months and percent changes in LS, TH, and FN BMD, as well as P1NP and TRACP-5b at six and 12 months. ISR was the independent variable, and percent changes in BMD were the dependent variables. Both univariate and multivariate models were constructed, with adjustments made for factors that showed statistically significant differences in the comparative analysis.

To address missing bone turnover marker data at 12 months (ISR group: n=17; non-ISR group: n=47), we conducted complete case analysis by excluding patients with missing values (case-wise deletion). All statistical tests were two-sided. A p-value of <0.050 was considered statistically significant. Analyses were performed using JMP® Pro version 17 (SAS Institute, Cary, NC, USA).

## Results

Baseline characteristics

A total of 181 patients were analyzed, with a mean age of 74.9 years (range: 52-100 years) (Table 1). Among these, 44 women (24.3%) experienced ISR within the first six months of romosozumab treatment. The baseline characteristics of the ISR and non-ISR groups are also summarized in Table 1. The mean age in the ISR group was significantly lower than that in the non-ISR group (72.2±7.7 years vs. 75.8±9.1 years, p=0.020). No other statistically significant differences in baseline characteristics were observed between the groups. The baseline imbalance in age may affect the interpretation of treatment effects in this comparative study.

**Table 1:** Baseline characteristics of the study cohort. ^a^: Continuous data are presented as mean±standard deviation and were compared using Student's t-test between the two groups. ^b^: Categorical data are presented as numbers (percentages) and were compared using Fisher’s exact test between the two groups. Test statistics (t values) are presented for continuous variables. For Fisher’s exact test, only p-values are reported as no test statistic is defined. ISR: injection-site reaction; BMD: bone mineral density; LS: lumbar spine; TH: total hip; FN: femoral neck; eGFR: estimated glomerular filtration rate; 25OHD: 25-hydroxyvitamin D; P1NP: procollagen type-1 N-terminal propeptide; TRACP-5b: tartrate-resistant acid phosphatase isoform 5b.

Table 1. Baseline characteristics of the study cohort.					
	Overall (n=181)	ISR group (n=44)	Non-ISR group (n=137)	Statistic	p-value
Age, years	74.9±8.9	72.2±7.7	75.8±9.1	t=–2.35	0.020^a^
Body mass index, kg/m^2^	21.2±3.2	20.7±2.9	21.4±3.2	t=–1.22	0.225^a^
Prior vertebral fracture, n(%)	75 (41.4)	13 (29.6)	62 (45.3)	–	0.079^b^
Prior non-vertebral fracture, n (%)	44 (24.3)	12 (27.3)	32 (23.4)	–	0.687^b^
LS BMD, g/cm^2^	0.74±0.14	0.70±0.13	0.75±0.14	t=–1.72	0.088^a^
TH BMD, g/cm^2^	0.62±0.09	0.62±0.09	0.61±0.09	t=0.22	0.829^a^
FN BMD, g/cm^2^	0.57±0.09	0.57±0.08	0.57±0.09	t=0.19	0.847^a^
eGFR, mL/min/1.73 m^2^	74.3±19.8	77.3±19.7	73.3±19.8	t=1.15	0.250^a^
Serum albumin, g/dL	4.2±0.3	4.3±0.3	4.2±0.3	t=1.68	0.095^a^
Serum-corrected calcium, mg/dL	9.0±0.4	9.0±0.4	9.0±0.4	t=–1.20	0.231^a^
25OHD, ng/mL	16.3±5.5	15.5±5.7	16.6±5.4	t=–1.20	0.231^a^
P1NP, μg/L	77.3±31.6	81.6±38.1	76.0±29.2	t=1.02	0.309^a^
TRACP-5b, mU/dL	613.6±216.4	631.6±211.7	607.8±218.4	t=0.63	0.526^a^

Main analysis

**Figure 1:**
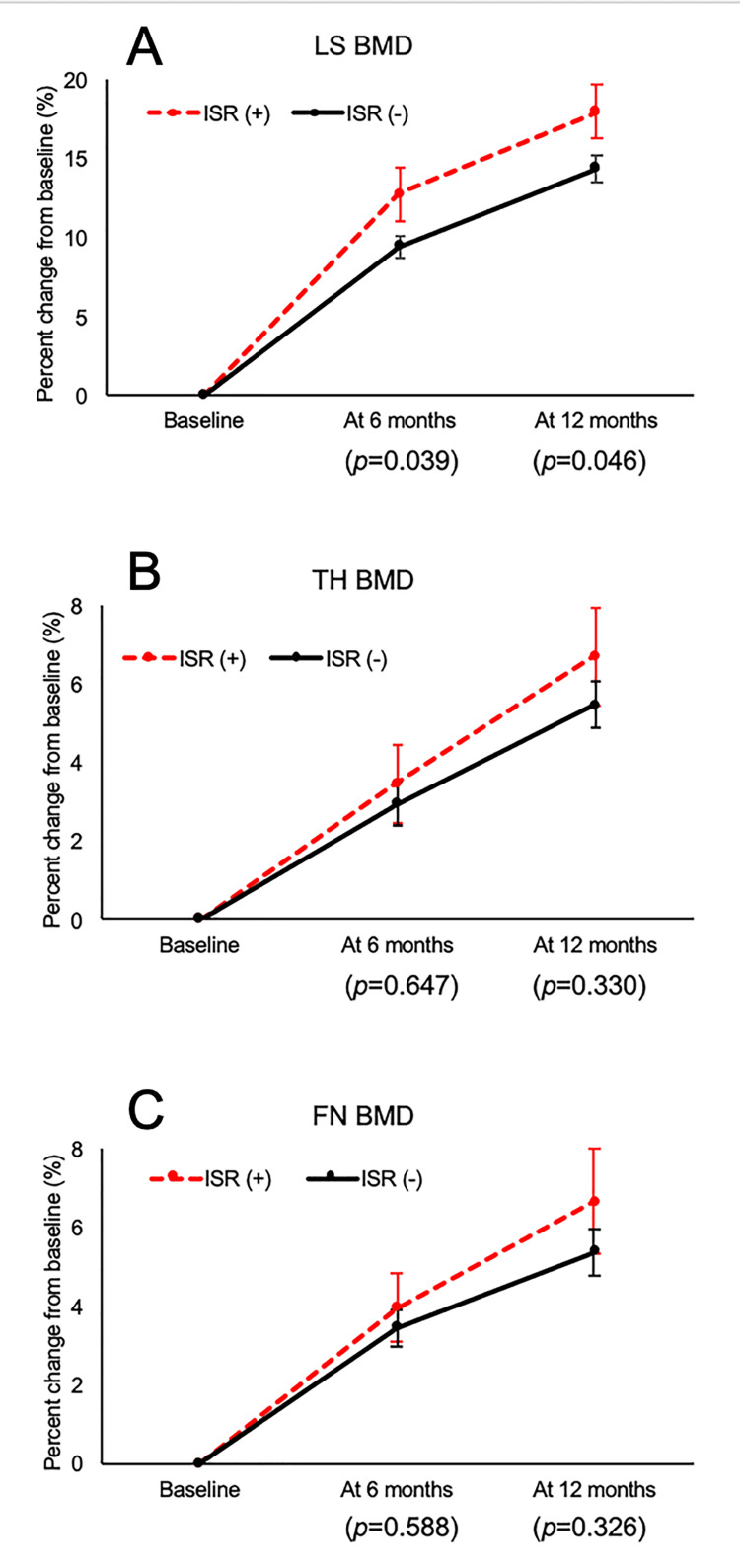
Mean percentage change in BMD at the LS (A), TH (B), and FN (C) from baseline to 6 and 12 months after treatment in the ISR and non-ISR groups. Bars indicate the mean±standard error. p-values from Student’s t-test are shown for differences between the two groups. ISR: injection-site reaction; BMD: bone mineral density; LS: lumbar spine; TH: total hip; FN: femoral neck.

**Figure 2:**
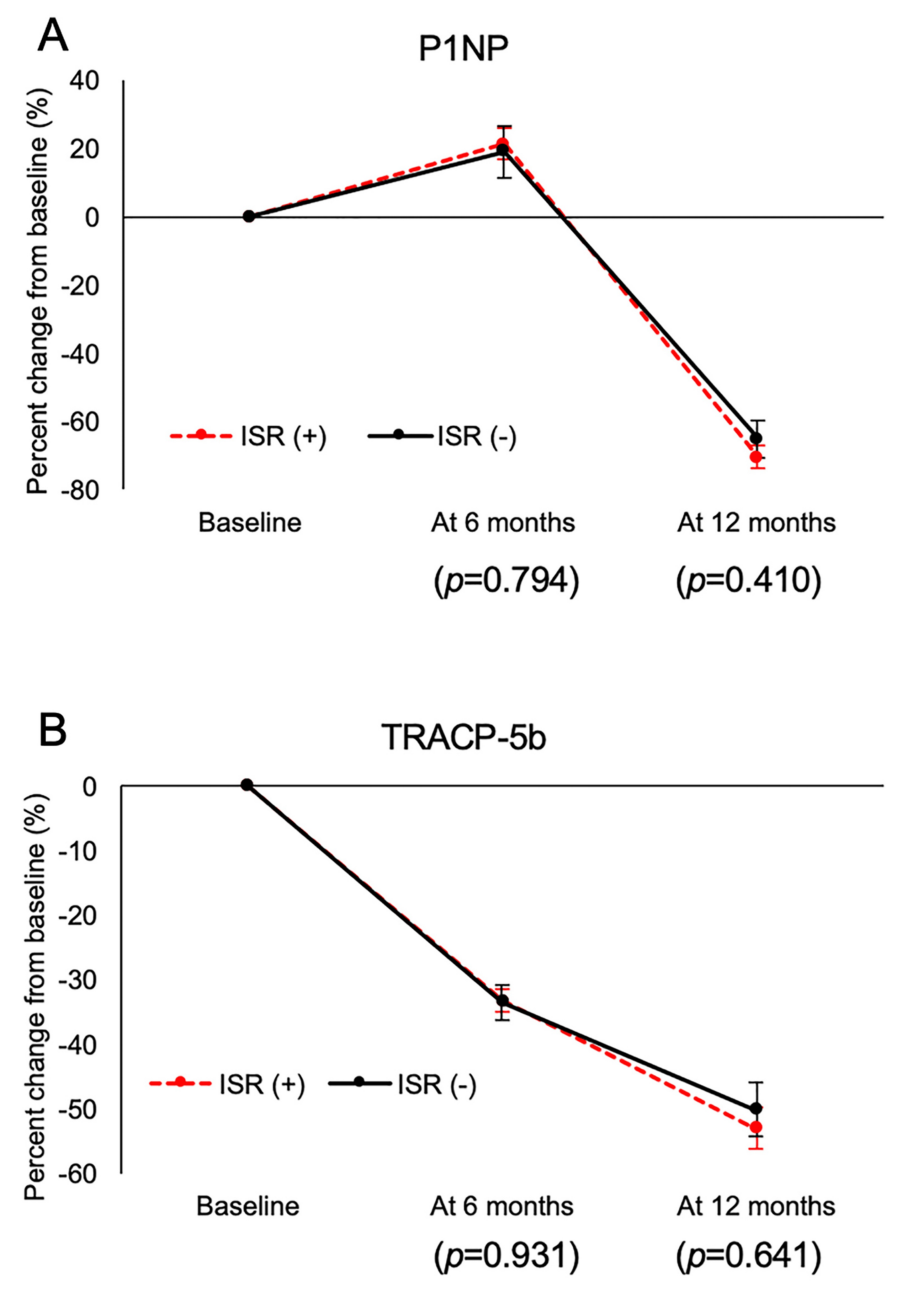
Mean percentage change in P1NP (A) and TRACP-5b (B) from baseline to 6 and 12 months. Bars indicate the mean±standard error. p-values from Student’s t-test are shown for differences between the two groups. Complete case analysis was used to address missing 12-month data (ISR group, n=17; non-ISR group, n=47). ISR: injection-site reaction; P1NP: procollagen type 1 N-terminal propeptide; TRACP-5b: tartrate-resistant acid phosphatase isoform 5b.

Mean percentage changes in LS, TH, and FN BMD from baseline to six and 12 months in both groups are shown in Figure 1. The ISR group exhibited significantly greater increases in LS BMD than the non-ISR group at both six months (12.8±11.4% vs. 9.5±8.4%, p=0.039) and 12 months (18.0±11.3% vs. 14.4±9.8%, p=0.046). In contrast, no significant differences were found between the ISR and non-ISR groups in the percent changes of TH BMD at either six months (3.5±6.5% vs. 2.9±6.7%, p=0.647) or 12 months (6.7±8.2% vs. 5.5±6.8%, p=0.330), or in FN BMD at six months (4.0±5.7% vs. 3.5±5.5%, p=0.588) or 12 months (6.7±8.8% vs. 5.4±6.9%, p=0.326). However, the ISR group consistently showed numerically greater increases in both TH and FN BMD at all time points, although these differences were not statistically significant. The percent changes in bone turnover markers, P1NP and TRACP-5b, did not differ significantly between the two groups at any time point (Figure 2). However, these results should be interpreted with caution because of missing data at 12 months.

Sensitivity analysis

To evaluate the robustness of the main findings, sensitivity analyses were conducted using linear mixed models (Table 2). In the univariate analysis, ISR within the first six months was significantly associated with greater percent increases in LS BMD at six months (estimate: -1.66, 95% confidence interval [CI]: -3.23 to -0.09, p=0.039) and 12 months (estimate: -1.77, 95% CI: -3.51 to -0.03, p=0.046). In the multivariate analysis adjusting for age, these associations were attenuated but remained marginally statistically significant. Age-adjustment did not alter the direction or magnitude of the estimates. No significant associations were found between ISR and percent changes in TH or FN BMD, or in P1NP and TRACP-5b, at any time point in either model. These sensitivity analyses support the robustness of the main findings, suggesting that the greater increases in BMD observed in the ISR group may have clinical relevance.

**Table 2:** Sensitivity analysis using linear mixed models to assess the robustness of the association between ISR and percent changes in BMD and bone turnover markers from baseline to 6 and 12 months. ISR: injection-site reaction; BMD: bone mineral density; LS: lumbar spine; TH: total hip; FN: femoral neck; P1NP: procollagen type-1 N-terminal propeptide; TRACP-5b: tartrate-resistant acid phosphatase isoform 5b; CI: confidence interval.

	Univariate estimate (95% CI)	p-value	Age-adjusted estimate (95% CI)	p-value
6-month percent change of LS BMD	–1.66 (–3.23, –0.09)	0.039	–1.37 (–2.95, 0.21)	0.089
12-month percent change of LS BMD	–1.77 (–3.51, –0.03)	0.046	–1.68 (–3.45, 0.09)	0.063
6-month percent change of TH BMD	–0.26 (–1.40, 0.87)	0.647	–0.24 (–1.40, 0.92)	0.680
12-month percent change of TH BMD	–0.61 (–1.82, 0.62)	0.330	–0.63 (–1.87, 0.62)	0.320
6-month percent change of FN BMD	–0.26 (–1.21, 0.69)	0.588	–0.33 (–1.29, 0.64)	0.507
12-month percent change of FN BMD	–0.63 (–1.91, 0.64)	0.326	–0.55 (–1.84, 0.75)	0.406
6-month percent change of P1NP	–1.15 (–9.79, 7.50)	0.794	–1.96 (–10.75, 6.84)	0.661
12-month percent change of P1NP	2.70 (–3.77, 9.18)	0.410	2.25 (–4.31, 8.80)	0.498
6-month percent change of TRACP-5b	–0.15 (–3.46, 3.17)	0.931	–0.38 (–3.76, 3.00)	0.824
12 -month percent change of TRACP-5b	1.46 (–4.72, 7.63)	0.641	0.99 (–5.26, 7.24)	0.754

## Discussion

This sub-analysis of a previously reported prospective cohort study investigated whether early ISRs within the first six months were associated with greater BMD increases following romosozumab treatment in untreated women with primary osteoporosis. We found that patients who experienced ISRs during the first six months of treatment had significantly greater increases in LS BMD at both six and 12 months compared to those without ISRs.

Romosozumab promotes bone formation and inhibits bone resorption by enhancing Wnt signaling through sclerostin inhibition [1,2]. Beyond its skeletal role, sclerostin is increasingly recognized as an immunomodulatory molecule, particularly through its interactions with immune cells such as B cells [26]. Interestingly, immune systems involving B cells may also regulate bone remodeling [27]. The concept of “immunoporosis” highlights the relationship between systemic immune status and bone metabolism [28]. Based on insights from osteoimmunology, we speculate that ISRs may not only reflect localized immune responses but also indicate a systemic immunological environment that enhances the skeletal response to romosozumab.

The potential association between ISRs and treatment response has been observed in other fields, such as vaccine responses, where ISRs following vaccination may indicate a stronger immune response [30]. Although ISR is typically considered an adverse event, it is also a clinically observable indicator of vaccine efficacy [30]. A similar concept may apply in the context of osteoporosis, where ISRs could reflect the therapeutic efficacy of romosozumab.

In this study, no significant group differences were observed in TH or FN BMD changes, but the ISR group consistently showed numerically greater increases in BMD across all sites. These findings may be explained by romosozumab’s particularly strong anabolic effect on trabecular bone, such as in the LS, while its effects on cortical-rich sites like the TH and FN are more modest [2-6]. Trabecular bone has a higher turnover rate and is therefore more responsive to romosozumab, whereas cortical bone remodels more slowly and shows a delayed response.

Bone turnover markers such as P1NP and TRACP-5b did not differ significantly between groups, although a detailed analysis was limited by incomplete follow-up data. Considering that the association between ISR and LS BMD gains was attenuated to marginal significance in the multivariate model, whether ISR serves as an independent predictor of treatment response remains unclear. Further research is needed to clarify this potential relationship.

In clinical practice, the presence of ISRs may be viewed positively by both clinicians and patients as a clinically observable indicator of treatment efficacy. Conversely, the absence of ISR should not prompt treatment discontinuation but rather support continued therapy, as the role of ISR as an independent predictor of treatment response remains uncertain.

Strengths and limitations of the present study

This study has several strengths. First, it offers a novel perspective by reframing ISRs, typically viewed as adverse events, as potential no-cost, clinically observable indicators of romosozumab efficacy that require no additional testing. Second, unlike previous studies involving heterogeneous populations [13-17], this study focused exclusively on treatment-naïve women with primary osteoporosis.

However, several limitations should be acknowledged. First, the sample size was limited, possibly contributing to non-significant findings despite numerical trends. The relatively limited sample size restricted the number of covariates that could be included in the multivariate models. Second, because of missing bone turnover marker data at 12 months in 64 patients, detailed evaluation of their association with ISRs was limited. The missingness affected not the primary outcome but the secondary exploratory outcomes. Third, although multivariate models adjusted for age, residual confounding (e.g., nutritional status, physical activity, medications) may still exist. Fourth, the study population was limited to Japanese women with primary osteoporosis, which may limit generalizability to other populations. Fifth, ISR ascertainment was unstandardized across five centres with no severity grading, making faithful reproduction difficult. Sixth, supplementation compliance was neither mandated nor reported, introducing an uncontrolled variable that affects BMD outcomes. Seventh, inter-center measurement variability could not be completely excluded. Finally, this study did not directly investigate the mechanistic pathways linking ISRs and BMD changes; therefore, the immunological basis of the observed association remains speculative.

## Conclusions

In this sub-analysis of a previously reported prospective cohort study of 181 untreated women with primary osteoporosis, those who experienced early ISRs within the first six months of romosozumab treatment showed significantly greater increases in LS BMD at both six and 12 months compared to those without ISRs. ISR could serve as a no-cost and clinically observable indicator associated with LS BMD response to romosozumab that requires no additional testing. Further studies are needed to validate this hypothesis and clarify the underlying mechanisms.
